# The person-based development and realist evaluation of a summary report for GP consultations [version 1; peer review: 2 approved]

**DOI:** 10.3310/nihropenres.13258.1

**Published:** 2022-02-21

**Authors:** Mairead Murphy, Geoff Wong, Anne Scott, Victoria Wilson, Chris Salisbury

**Affiliations:** 1 Bristol Medical School, University of Bristol, Bristol, BS8 2PS, UK; 2 Nuffield Department of Primary Care Health Sciences, University of Oxford, Oxford, OX2 6GG, UK

**Keywords:** GP consultations, GP-patient communication, person-based approach, realist evaluation

## Abstract

**Background:**

Use of telephone, video and e-consultations is increasing. These can make consultations more transactional. This study aimed to develop a complex intervention to address patients’ concerns more comprehensively in general practice and test the feasibility of this in a cluster-randomised framework.

The complex intervention used two technologies: a patient-completed pre-consultation form used at consultation opening and a doctor-provided summary report provided at consultation closure. This paper reports on the development and realist evaluation of the summary report.

**Methods:**

A person-based approach was used to develop the summary report. An electronic protocol was designed to automatically generate the report after GPs complete a clinical template in the patient record. This was tested with 45 patients in 3 rounds each, with iterative adjustments made based on feedback after each round. Subsequently, an intervention incorporating the pre-consultation form with the summary report was then tested in a cluster-randomised framework with 30 patients per practice in six practices: four randomised to intervention, and two to control. An embedded realist evaluation was carried out. The main feasibility study results are reported elsewhere.

**Results:**

**Intervention Development**

15 patients were recruited per practice. Eight patients and six GPs were interviewed and 18 changes made. The summary report improved substantially; GPs and patients in the final practice were more satisfied with the report than the first practice.

**Realist evaluation**

The summary was most useful for consultations when safety-netting advice was important or with multiple complex follow-up steps in patients who have difficulty remembering or communicating. It generated greater clarity on the follow-up and greater patient empowerment and reassurance.

**Conclusions:**

The person-based approach was successful. The summary report creates clarity, empowerment and reassurance in certain consultations and patients. As it takes a few minutes per patient, GPs prefer to select patients who will benefit most.

## 1 Background

### 1.1 Rationale for study

The Calgary-Cambridge guide, which is used as a basis for training medical students and doctors, identifies six steps to conducting a GP consultation: initiating, information gathering, providing structure, relationship building, explanation/planning and closing^1^. Opportunities to address patients problems are commonly missed at consultation initiation (when the GP should elicit the patient’s reason for attendance)^2^. Problems can remain unaddressed at consultation closure, if advice given is unclear, particularly with regards advising patients what to do if the problem does not resolve, or gets worse, which GPs refer to as “safety-netting”^3^. Research suggests that interventions at each end of the consultation can help to address patient concerns. At consultation initiation, sharing the results from patient-reported outcome measures (PROMs) with clinicians can help to elicit concerns^4^. At consultation closure, providing the patient with written as well as spoken information can improve recall and adherence^5^.

To help with this problem, we designed an intervention: the Consultation Open and Close (COAC) intervention which used a pre-consultation form at consultation opening and a summary report at consultation closure. We then tested these interventions in a feasibility study.

### 1.2 Provision of written information at consultation closure

The closing steps of a consultation are when clinicians summarise, make a plan with the patient, safety-net and check the patient’s understanding^6^. Patients often raise last-minute concerns at this point, particularly if all concerns have not been elicited early on^7^. Patients’ memory for advice on treatment and follow-up after the consultation tends to be worse with older people; if the information given contradicts existing beliefs; or if potentially life-altering diagnostic information is given^5^.

Written advice can be provided at any point in the consultation but is most often provided at consultation closure. This may be general information on a specific condition, healthy lifestyle advice or safety-netting advice. Provision of written information can improve patient understanding, memory of the consultation and subsequent adherence^5,8^. Patients remember specific advice which is individually tailored to them more easily than generic advice provided in patient information leaflets^5^. Where patients are routinely provided with information on their medication and consultation, through direct patient record access, this has improved patient safety and adherence^9^.

The aim of this study was to develop and test an intervention to more comprehensively address patients’ concerns in general practice through use of a pre-consultation form at consultation opening and a summary report on consultation closure.

This paper describes the development of the summary report and a realist evaluation of its use from the feasibility study of the COAC intervention. The development and testing of the pre-consultation form and the full feasibility study results are reported separately in two papers published alongside this one.

## 2 Methods

### 2.1 Study setting

This study was based in primary care involving general practices serving different patient populations in Bristol, North Somerset and South Gloucestershire (BNSSG). Practices were selected from areas within a range of socioeconomic deprivation levels as well as urban, suburban and rural areas.

The COVID-19 pandemic occurred six months into this study. Under an NIHR directive, the study was paused in March 2020 and restarted in September 2020. Research protocols were updated so the intervention and research did not require face-to-face contact.

### 2.2 The Consultation Open and Close (COAC) Study

The COAC Study involved the development and testing of an intervention, incorporating use of an individual-level PROM at consultation opening and written information at consultation closure. The primary aim of the COAC study was to develop and test the feasibility of a complex intervention designed to more comprehensively address patients’ concerns in general practice, thereby reducing re-consultation rates, improving patients’ well-being and health knowledge, reducing health concerns and increasing patients’ confidence in their health provision and health plan.

The COAC study incorporated two phases: an Intervention Development study (Study 1) and a feasibility study (Study 2) as follows:

***Intervention Development Study:*** This involved design of a complex intervention to improve the ability of GPs or nurse practitioners to address patients’ concerns by a) incorporating the use of an electronic patient questionnaire consultation opening and b) providing a summary report at consultation closure, which is either printed or texted to the patient or is accessible from the patient record. These were designed and evaluated separately, in accordance with MRC guidance for design of complex interventions^10^.

***Feasibility Study:*** In this study, the COAC intervention was tested in a cluster-randomised framework to establish the feasibility of both a randomised-control trial of the intervention and the intervention itself.

**Figure 1:**
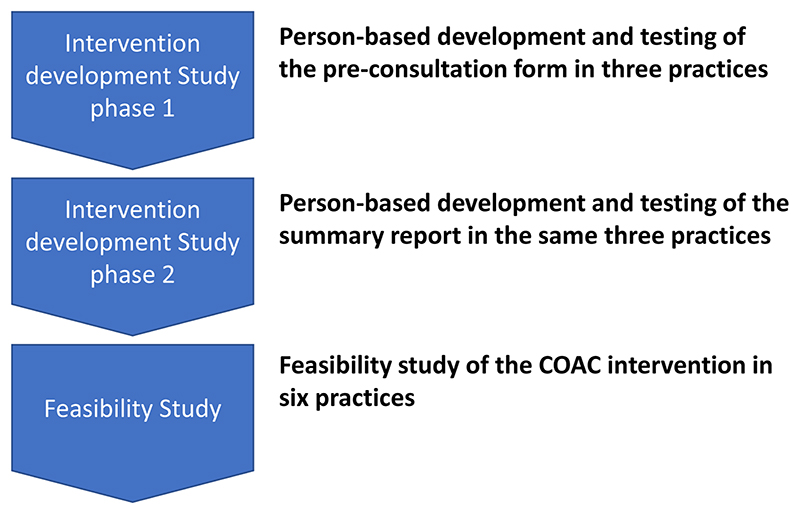
The Intervention Development and Feasibility Studies.

The sequential nature of the studies is shown in Figure 1.

### 2.3 Recruitment

#### 2.3.1 Practice recruitment

Practices were approached by the NIHR Clinical Research Network for the West of England (hereafter referred to as the CRN) with the information on the study. Practices were recruited to the two phases separately, with practices who participated in the Intervention Development study actively encouraged to continue their participation in the Feasibility study.

For each study, the CRN shared the Research Information Sheet for Practices (RISP) which was developed for the study with a range of practices meeting the inclusion criteria. Interested practices then contacted the study chief investigator (CI) who arranged a meeting(s) with the practice manager or GP research lead.

Practice representatives were asked to sign a practice agreement consenting to the practice taking part in the study. Practices were approached for Study 1 in November 2019 (three practices were required for Study 1); and for Study 2 in May 2021 (six practices were required for Study 2).

All selected practices already used SMS software (MJOG and accuRx) and the patient record system EMIS. Administrators were expected to be familiar with the process of sending batch texts using practices SMS software (e.g. MJOG) and in uploading reports to and setting alerts in EMIS.

#### 2.3.2 Eligibility criteria

For the Intervention Development study (three practices) we purposively selected: one practice in the top deprivation quartile, one at the median, and one in the lower quartile. For the feasibility study (six practices), we selected three practices in the top two deprivation quartiles and three practices in the bottom two.

Patients in the intervention development study were recruited to receive a summary report by GPs if they were:

▪ Aged 17 or over (on date of SMS invitation to participate)
▪ Had an appointment with that GP and the GP thought a summary report would be useful
▪ The patient agreed a summary report would be useful

In the feasibility study patients were recruited using these three same criteria, but additionally only if they had already completed a pre-consultation form.

The remainder of the methods are described separately for the Intervention Development study and feasibility study in the respective two sections that follow.

### 2.4 Intervention development study methods

#### 2.4.1 Approach

**Figure 2:**
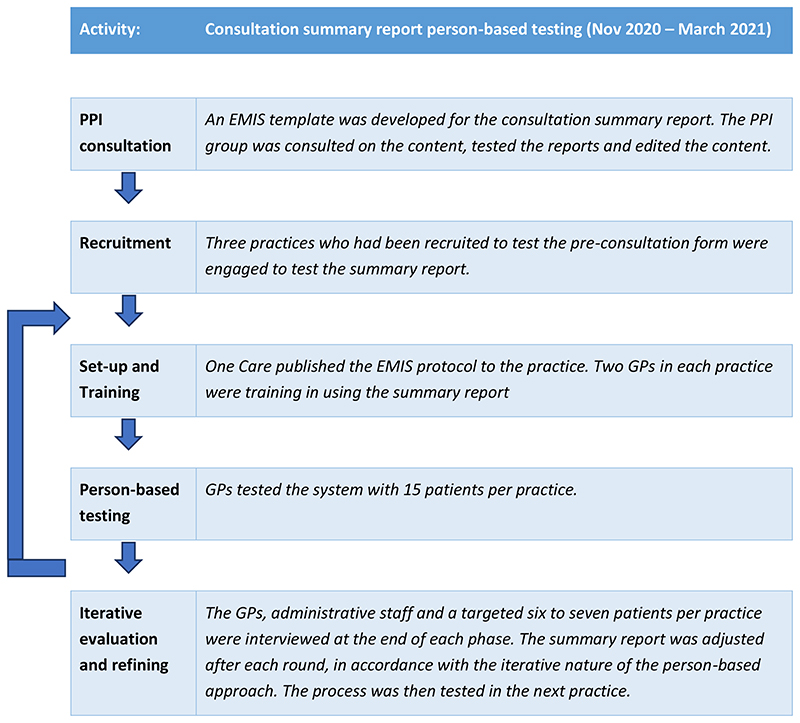
Person-based approach taken to develop the summary report.

The Intervention Development Study was carried out in two distinct parts, one for development of the online pre-consultation form and one for development of the summary report provided at consultation closure. Development of the summary report is described in this section. A prototype was developed based on the research literature and a series of patient and public involvement (PPI) group consultations. This was then tested with actual patients using a person-based approach, which involves using mixed-methods research to systematically investigate the needs, attitudes and situation of the people who will be using the intervention^11^. Through the person-based approach, each step of the intervention was tested in rounds and adjusted after each round according to the feedback given from patients and clinicians. This iterative approach is shown in Figure 2.

#### 2.4.2 Prototype development

##### Starting position

Based on consultation with our PPI group we designed a report with four sub-headings as follows:

1. Issues raised in the consultation today
2. Advice given
3. Treatment
4. Follow-up and safety netting

One Care, the Federation for GP Practices in BNSSG, developed an EMIS protocol, clinical template and document template that GPs could use to generate this report. EMIS is the GP patient record system used in BNSSG. The protocol allowed GPs to load a clinical template (which is a structured form) to allow them to input information under the four headings above. When the GP saved the template, a Microsoft Word report was automatically loaded that could be saved to the record and printed or sent to patients.

##### Initial programme theory

**Figure 3:**
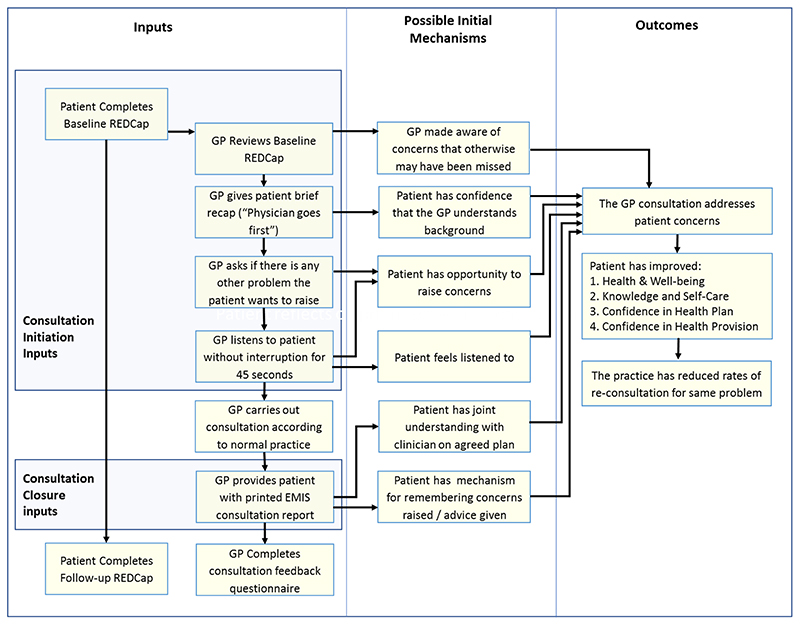
Proposed initial programme theory of COAC.

An initial programme theory of how the COAC intervention was intended to produce outcomes was designed. This was drafted by the study CI and reviewed by the study co-applicants and PPI group before finalisation. This is shown in Figure 3.

#### 2.4.3 Patient recruitment

Patients were recruited to the intervention development study by GPs at the end of the consultation. The recruiting GPs explained to the patients that they were trying out providing a summary report of the consultation and asked the patient if they would like to receive this report. Along with the summary report, the clinician gave the patient an information leaflet about the study, with the researcher contact details. Patients were asked to contact the researcher if they were willing to be interviewed. In case the patients did not contact the researcher, a follow-up text was sent to all participating patients who received the patient information leaflet, reminding patients of the request to interview and asking them to respond “OK” if they were happy for their contact details to be shared with the researcher. The researcher then contacted these patients for interview.

#### 2.4.4 Data collection / measures

Data collected in the intervention development study included clinician and administrator questionnaire data and qualitative interviews. Interviews were carried out by MM and AS and audio recorded. We aimed for 20 patient interviews, 6 GP interviews and 3 administrator interviews.

The purpose of these interviews was to inform development of the intervention through a person-based approach (which takes place in rounds, with the intervention changed at the end of each round). Topic guides therefore focussed on the feasibility and perceived usefulness of summary report, and on the proposed design of the intervention. Patient and GP topic guides are available as open access data (see data availability statement).

Prior to the COVID-19 pandemic, some interviews were conducted face-to-face in the patients’ own homes or other location of their choice, and GP/administrator interviews in the health centre. After March 2020 interviews were conducted by telephone or video link.

#### 2.4.5 Analysis

Interviews were transcribed and analysed at the end of each round. The analysis focussed on establishing what changes were required to the summary report in that round before testing again in the next practice.

To do this, “guiding principles” were established. These are fundamental to the person-based approach, and highlight the objectives of the intervention and the key features that will address context-specific behavioural issues in support of these objectives^11^. The guiding principles were drafted by the CI, adjusted by the PPI group and agreed by the study co-investigators. A coding framework for changes identified was then established (see Table 1). This framework contained codes to identify the reason for making each change, with reference to the guiding principles and the initial programme theory. After each interview was transcribed, one of two researchers (MM or AS) listed the possible changes arising from that interview and assigned a code to it. The other researcher then checked this and, if necessary, added new changes to the list or modified existing ones. The two researchers then discussed any areas of disagreement.

At the end of each round, the co-applicants all reviewed the table of changes and a final list for the round was agreed. The changes were implemented and the revised summary report was taken forward to the next round.

This continued for three rounds until a final version of the summary report was agreed.

### 2.5 Patient and Public Involvement

This research was informed by Patient and Public Involvement (PPI) both before the study commenced and during the study. PPI contributors received expenses and reimbursement in line with INVOLVE guidance^12^.

The PPI group helped draft the person-based guiding principles, agreed the initial programme theory and made significant contributions to the structure, wording and look of both the pre-consultation questionnaire and the closure summary report. The group then met five times throughout the study. Two of these meetings were specifically to design the summary report as follows:

▪ A meeting was convened for members to give input on the consultation summary report before the person-based development of this started. The meeting resulted in substantial wording changes to the first version of the report and in changing the format from 4 sections to 2. (See section 3.1.2).
▪ Another meeting was convened to solicit additional PPI input on the detailed wording of consultation summary report due to a difficulty in eliciting this from the patient interviews in the second round. In this meeting, members developed a number of wording options for specific areas of the form.
▪ Following this meeting, an online consultation process took place whereby PPI members selected their preferred choice from the wording options designed at the previous meeting. The GPs in round 3 of the intervention development phase also voted on these and commented on the PPI group’s selection.

The PPI group also met at the end of the feasibility study to comment on the overall interpretation of the data and discussed how the results could be used in the future, either for additional research or how to benefit patients and clinicians in the future. The group assisted with drafting and approving the Plain English Summary for this paper and other publications.

### 2.6 Feasibility study methods

To provide some context for the realist evaluation, brief details are provided in this paper on the randomisation, recruitment and consent and data collection / measures of the feasibility study. This section mainly focusses on describing the data and analysis used for the realist evaluation embedded within the feasibility study^13^.

#### 2.6.1 Randomisation and recruitment

In the feasibility study, both the pre-consultation form and summary report were used together in six practices, four randomised to intervention and two to control. Practices who had participated in the Intervention Development study were approached by the CI and new practices were approached by the CRN. Each practice was asked to recruit 30 patients (see Table 2) but GPs were asked to complete a summary report for patients only if it was useful. Because of the workload pressure GPs were under due to the COVID-19 pandemic, they were not given a specific target but were told that this could be more than 50% of patients or as few as 20%. Based on the intervention development study, the researcher expected an estimated 30% to receive a closure report.

Patients were recruited to the COAC intervention via an SMS message which contained a link for them to access the pre-consultation form. Patients who completed this form had it uploaded to the record by administrators so GPs could see it at the start of the consultation. During the consultation, GPs decided whether to give each patient a summary report. GPs were not given prescriptive criteria but advised that the form might be most useful when:

▪ They had ordered diagnostic tests for the patient
▪ They had made a referral
▪ They had given the patient important safety-netting advice
▪ They had made complex changes to the patient’s medication

However, GPs were advised that the decision on whether to offer a report was an individual decision to be made by the GP on consultation with the patient.

#### 2.6.2 Data collection / measures

Feasibility study data included clinician and administrator questionnaire data, interview data, and quantitative patient data. The quantitative patient data is described in the feasibility study linked paper^13^. Interview and questionnaire data were collected as follows:

**Clinician questionnaire data:** GPs were asked to complete a questionnaire row for each recruited patient. Within this questionnaire there was a question asking if clinicians had used a summary report and, if so, the reasons for this.

**Interview data:** Interviews in the feasibility study were conducted by MM and AS. Topic guides were designed to inform a realist evaluation and therefore focussed on the outcomes that patients/GPs perceived, the mechanisms by which these were achieved and the contexts. We aimed to interview patients and practitioners to the point of achieving “theoretical sufficiency”, i.e. when the data analysis has yielded one or more coherent theories which are relevant to the study aims^14^. Interviews were conducted by phone and audio-recorded.

#### 2.6.3 Analysis

Realist evaluation seeks to explain the complex relationship between context, mechanisms and outcome. The explanatory proposition of realist evaluation is that interventions work (i.e. have successful outcomes) only in so far as the individuals involved take up ideas and opportunities (mechanisms) within the social and practical conditions in which they are operating (contexts)^15^. This is then reported in terms of contextual factors (What elements of the intervention work, for whom, in what consultations?) and content-mechanism-outcome configurations (CMOCs): a CMOC is a hypothesis that the program works to produce an outcome (O) because of the action of some underlying mechanism (M), which only comes into operation in particular contexts. (C)

The realist evaluation used the interview data collected in the feasibility study, supplemented with the interviews from the intervention development study. To carry out the realist evaluation, the CI (MM) read and re-read the initial interview transcripts from both patients and practitioners, in order to gain an overall view of the accounts given and to identify patterns in the data. She then revised the programme theory and devised an initial set of CMOCs. The research associate (AS) independently developed three lists of context, mechanisms and outcomes. These were cross-checked against the CI’s CMOCs which were then revised and detailed evidence presented against each of them. An experienced realist evaluator (GW), then read through the detailed evidence and the final CMOCs were agreed in collaboration. Four researchers (MM/AS/GW/CS) reviewed the realist evaluations findings and programme theory before finalising.

### 2.7 Consent

For the intervention development study patients gave consent to the GP to receive the intervention, and these patients contacted the researcher if they were happy to be interviewed. For the feasibility study, return of the questionnaire indicated consent to participate in the study. Patients were explicitly asked to consent to their contact phone number being shared with the University of Bristol for the purposes of sending a follow-up questionnaire. Further consent for use of that phone number to contact the patient for interview and for access to the patient’s record for demographics and re-consultation rates was requested in the follow-up questionnaire^16^. Patients were contacted for interview using the phone number they provided

For both the intervention development and feasibility study the researcher took informed consent for recording the interviews and use of anonymised quotations in publications prior to the interview itself. This consent was written for face-to-face interviews and audio-recorded for telephone interviews. Before the start of the COVID-19 pandemic, all consent was written. The ethics committee approved an amendment to collect audio-recorded consent for patients interviewed during the pandemic.

### 2.8 Sponsorship, funding and ethical arrangements

This study was sponsored by the University of Bristol. Ethics approval was granted by Frenchay Research Ethics committee^17^ and the Heath Research Authority (HRA). BNSSG Clinical Commissioning Group Research and Evidence Team provided research and development approval. The study was NIHR funded and supported by the NIHR Clinical Research Network who liaised with centres on the researchers’ behalf.

Insurance was provided by the University of Bristol as research sponsor. The study sponsor and funders did not have any role in study design; data collection, management, analysis, and interpretation of data; writing of the report; or the decision to submit the report for publication.

The Feasibility study was registered in the ISCTRN registry (ISRCTN13471877) and on the CRN portfolio (42005). The study protocol was published before recruitment completed^18^.

## 3 Results

### 3.1 Intervention development

#### 3.1.1 Participants

Table 3 shows the number of recruits to the Intervention Development study. Three practices were recruited from the top, middle and bottom of the index of deprivation (IMD) score, where 1 indicates a high level of deprivation and 10 a high level of affluence. Each practice had two participating GPs. One of these was an advanced nurse practitioner, but for simplicity and anonymity has been referred to throughout as a GP. 43 out of a target of 45 patients were recruited by GPs. We had intended to interview 20 patients from the 43 recruited but were only able to interview eight, as most patients did not contact the researchers for interview or respond “OK” to the text message asking if their details could be shared with the researcher. We therefore carried out an extended PPI consultation after the second round to augment the patient view.

The patient identifiers used herein are consistent with the feasibility study linked paper^13^, which has 50 unique patient identifiers. This is why some patient identifiers are higher in number than the overall number of patients we have data for (e.g. Patient 40).

#### 3.1.2 Summary of changes

The person-based approach relies on a set of guiding principles. These were agreed in advance and informed the intervention development by highlighting the objectives of the intervention and the key features that will address context-specific behavioural issues in support of these objectives^11^. (see Table 4)

Table 5 summarises the key elements of the consultation summary report that were changed over the 3 rounds. The full table of 18 changes, including verbatim quotes and coded rationale for making each change open access data (see data availability statement). As shown in Table 5, early versions of the summary report were presented in paragraph text. This was changed to a bulleted list which was easier for patients to read. The scope of the template was extended and it was made simpler for GPs to complete, with more prefilled tick-box text, which was customised for each practice. A structured PPI process was carried out to agree the details of the wording.

The process for generating the summary report changed substantially during the intervention development study. In the first version, GPs were provided with three different templates and selected one of these depending on whether they were ordering a diagnostic test, more than one test, or no tests. In contrast, the final version used a single template. GPs found this easier to use, because they only had get used to the look and feel of one form, and because they did not have to decide how many tests to order before opening that form. The first version also required a lot of free text entry, particularly for the “more than one test” template. In the final version, much of the report was generated by pre-filled tick-boxes, rather than free-text entry. This was quicker for GPs to complete.

A GP from site 1 (who were the first site to participate in the Intervention Development study and randomised to intervention in the Feasibility Study) commented that these changes were a substantial improvement.

I think the closure [summary report] is definitely better than last time. It looks much neater. It’s much easier to fill in […] I’d definitely consider using it in people that have got complex, multiple problems to give them that information. Whereas before, I think I would have said, ‘This is far too difficult. I don’t want to do this.’ **(GP 1 – Feasibility Study)**

This GP had felt during round 1 of the intervention development study that the pilot version of the summary report was too difficult to complete and she would not be happy to use it regularly. However, the final version used in the Feasibility Study was “much easier” to complete and she would be interested in using it with a subset of her patients.

**Figure 4:**
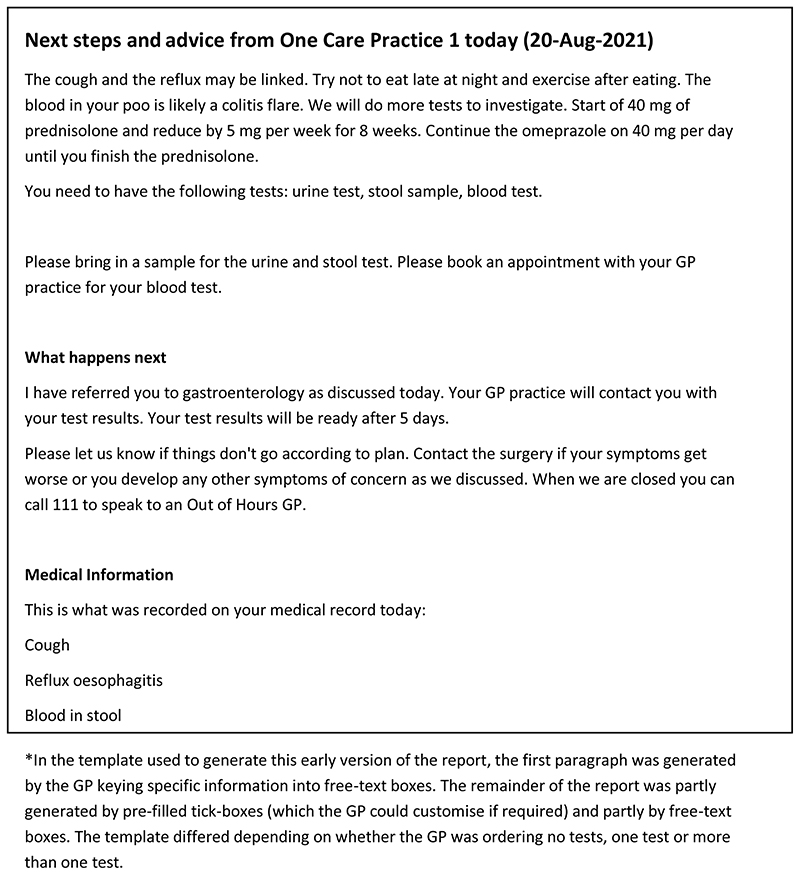
Consultation summary report: Pilot version (start of intervention development)*.

**Figure 5:**
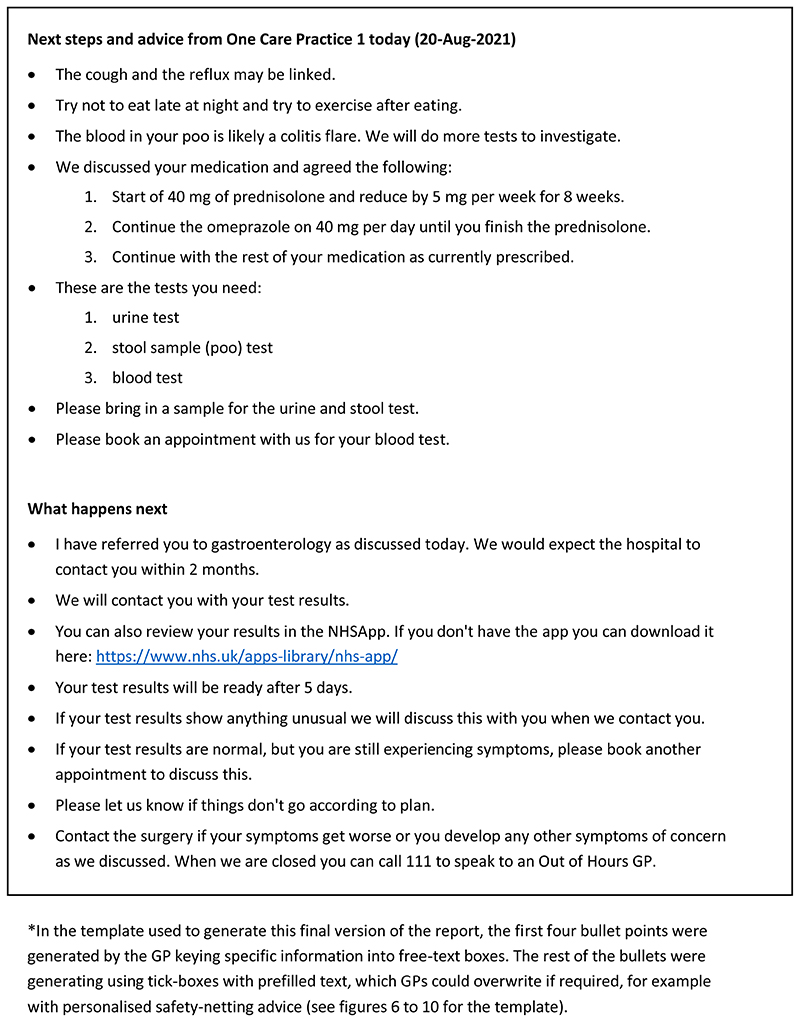
Consultation summary report: Final version (end of intervention development) *.

Examples of the original and the final versions of the consultation summary report is shown in Figure 4 and Figure 5 respectively.

**Figure 6:**
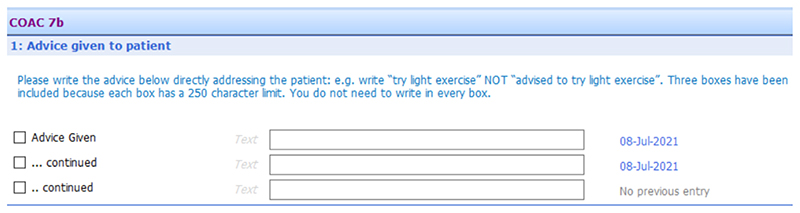
Summary report EMIS template – general advice section (1).

**Figure 7:**
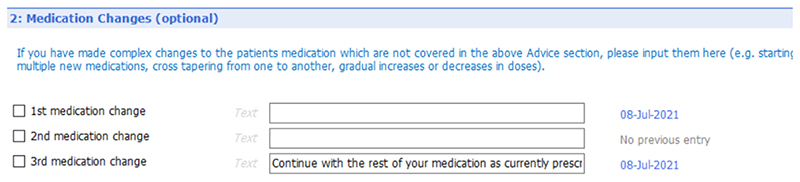
Summary report EMIS template – medication changes section (2).

**Figure 8:**
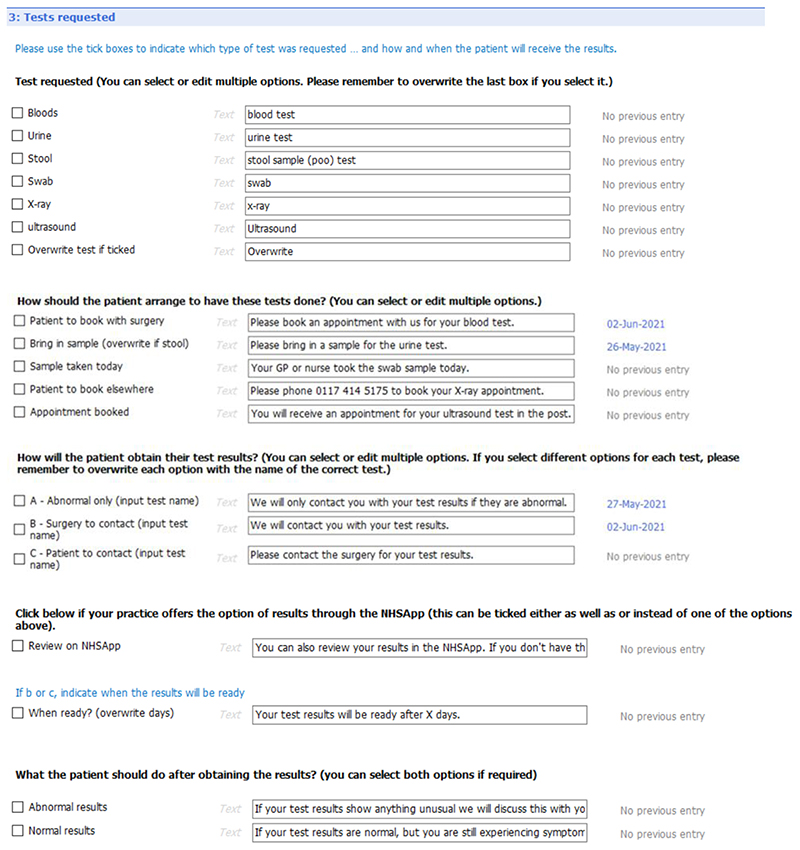
Summary report EMIS template – test requested section (3).

**Figure 9:**
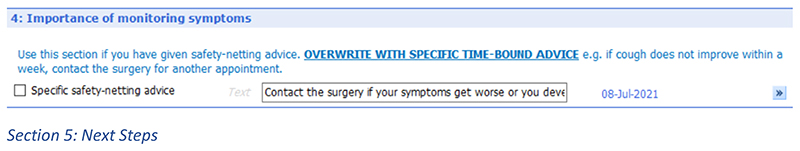
Summary report EMIS template – safety netting section (4).

**Figure 10:**
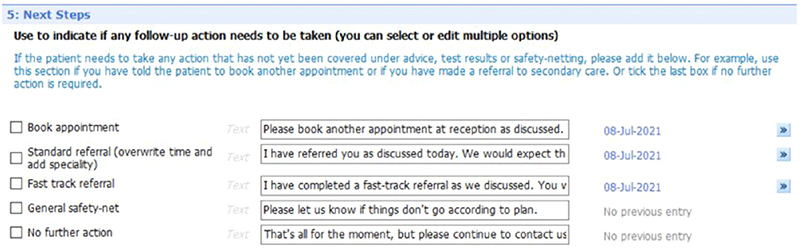
Summary report EMIS template – next steps section (5).

Figure 6 to Figure 10 show the five sections of the final EMIS form from the GP point of view, including where the form is pre-populated with standard adjustable text. Once the GP has decided to give the patient a summary report they launch the EMIS template from a shortcut key on their desktop. The EMIS template then loads on a single scrollable screen. The first section of this screen (Figure 6: advice given to patient) has three boxes, each for the GP to add a separate piece of advice. This first of these boxes is the only mandatory section in the template. The next section (Figure 7: medication changes) is for complex changes in medication and has two free text boxes, followed by a tick box containing the text “Continue with the rest of your medication as currently prescribed”. The third, and longest, section is for tests ordered (Figure 8: Tests ordered). In this section the GP can tick the names of the tests ordered, how the patient should arrange to get the tests done (pre-populated with the standard procedure for that GP practice), when and how the patient should expect to get the results and what to do if the results are abnormal or normal. The fourth section (Figure 9: safety-netting) is a single box for safety netting, populated with a standard statement that the GP can add more detail to if required. The final section (Figure 10: Next Steps) is for any other action, for example if the patient needs to book an appointment or a referral has been made.

**Figure 11:**
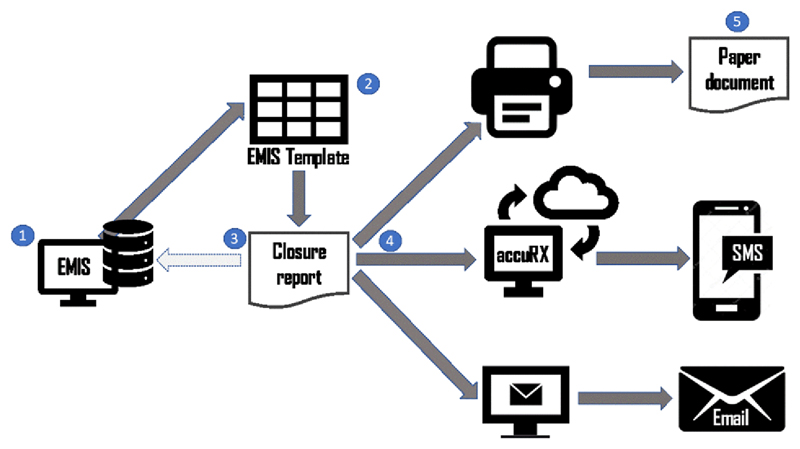
Consultation summary report generation: process diagram.

The final agreed process of generating the consultation summary report and providing it to the patient is shown in Figure 11. The steps shown in this figure are as follows:

1. **The GP** launches the COAC protocol from EMIS (This is done by pressing function->F12 and clicking “COAC protocol” in the window that appears.)
2. The template shown in section 3.2.5 is loaded onto the GP screen. **The GP** completes the five sections. On saving, the codes get written to the patient record and the summary report is generated.
3. **The GP** clicks on the summary report, which will open as a word document within the EMIS window. The GP presses Alt->G on the keyboard to run the macro to format the summary report. They make any other changes if they are required and save the report.
4. Now **the GP** has three options:
  - If the appointment is face to face, they can print the report and hand to the patient.
  - They can send it to the patient via accuRX
  - They can task an administrator to send to the patient it via email. Some patients preferred this, as the report is easier to see on email than SMS, but GPs preferred accuRX.
5. **The patient** receives the report. Depending on how the report was sent the patient correspondingly receives it via EITHER:
  - Paper copy
  - SMS
  - Email

### 3.2 Realist evaluation

#### 3.2.1 Participants

Thirty-two interviews were analysed. These interviews came from the following sources:

▪ **Intervention development study:** Eight patient interviews and six GP interviews.
▪ **Feasibility study:** Eleven patient and seven GP interviews.

Although we did not interview as many patients and practitioners as we had anticipated, the view of interviewees converged substantially, so we had sufficient “information power”^19^ to derive the new programme theory (see section 3.2.3).

The administrator interviews were not relevant for the summary report so were not included in the analysis. Interviews at each site in each phase are shown in Table 6.

In the analysis which follows, Patients 1 to 20 are from the intervention development study and patients 30 to 50 from the Feasibility study. So that the evolution of their views can be compared, the same identifier is used across the studies for GPs who were in both studies.

#### 3.2.2 Summary findings from the process evaluation

The process evaluation of the feasibility study is presented in the linked paper^13^. A key finding of this evaluation was that the pre-consultation form and summary report are useful for different types of patients and consultation and each intervention results in different outcomes, triggered via separate mechanisms. It was therefore more appropriate to carry out a separate realist evaluation for the pre-consultation form and the summary report respectively than to update the initial joint programme theory which was shown in Figure 3.

#### 3.2.3 Revised programme theory

**Figure 12:**
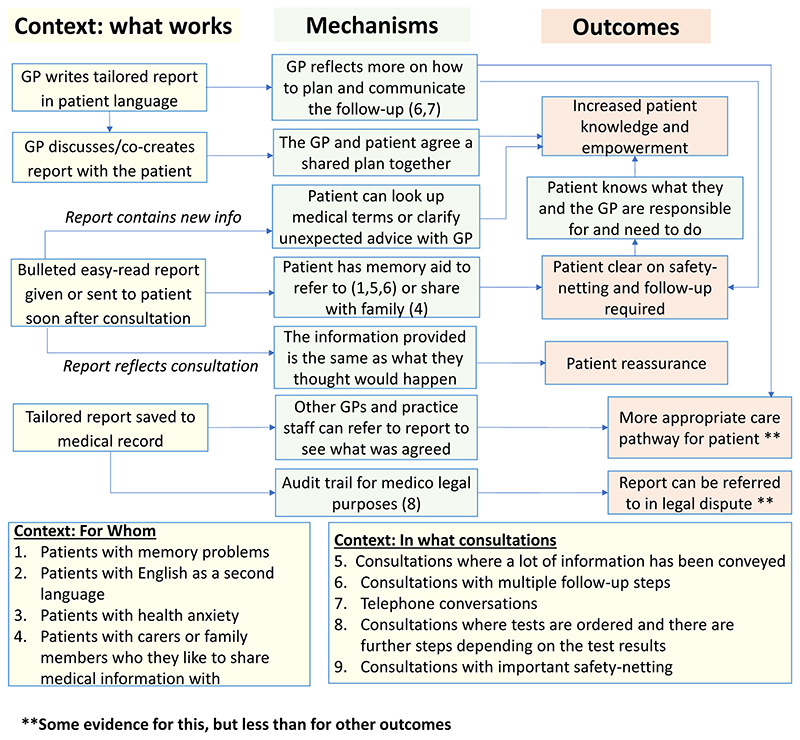
summary report, revised programme theory.

Our analysis of the data from 32 interviews enabled us to revise the programme theory for the summary report. This is shown in Figure 12. This presents nine context-mechanism-outcome configurations (CMOCs) identified in the data in a single diagram showing interlinked context (what works), mechanisms and outcomes. Context is characterised differently across realist evaluations^20^. We have interpreted context as anything in the environmental which interacts with (or ‘triggers’) mechanisms that cause an outcome of interest that we have identified in our programme theory. Interventions are deliberately designed so that they change those aspects of context that are modifiable which in turn will ‘trigger’ the relevant mechanism(s) needed to produce a desired outcome. Examples of context captured in the programme theory include “what works” (how the interventional subcomponent needs to be implemented to alter context and thereby trigger mechanisms to achieve desired outcomes), “for whom” (contextual aspects of the patient that interact with mechanisms) and “in what circumstances” (contextual aspects of the presenting problem or consultation that interact with mechanisms). The mechanisms are processes that are ‘triggered’ by the context to cause outcomes. Some mechanisms are only activated for certain types of patients and consultations. This information on which types of patients and consultations are shown by the numbers in brackets in the green mechanism boxes. One of the outcomes is more distal than the others and for this, the context in which they are achieved is represented by another outcome in the programme theory (functioning as a context for that CMOC).)

#### 3.2.4 Context (what works, for whom and what circumstances)

This section focuses on providing an overview of the key contexts which are needed for mechanisms to be activated. Details about the CMOCs in which these contexts function may be found in below in section 3.2.5.

##### 3.2.4.1 What works

Key elements of the summary report part of the COAC intervention that worked well were:

1)The GP writing tailored advice in patient language 2) the GP discussing / co-creating the report with the patient 3) the bulleted format sent to patients soon after the consultation 4) the report being saved directly to the patient record.

The summary report worked best when GPs provided tailored advice to patients. The quotes below are from two patients who received the report from two different GPs in the same practice. GP1 tended to write more specific advice and GP2 briefer advice:

I think considering how many different things we talked about, I think it was very thorough and very useful. (Patient 14, Intervention Development, Round 1)

The advice that she’s put on here isn’t very clear at all. The advice on here is one, two lines. It’s very basic information. (Patient 15, Intervention Development, Round 1)

Patient 14 found her report clear. The GP had ticked the generic box “The surgery will contact you if your blood tests are abnormal” but added tailored safety-netting advice. Patient 15 found the advice unclear. His GP had provided only brief advice. This was one of only two patients who did not find the summary report helpful; he was recruited in round 1 of intervention development before multiple improvements were made to the template.

The other three elements that worked well are discussed within the individual CMOCs they apply to.

##### 3.2.4.2 For what type of patient

GPs selected patients who might have problems remembering medical advice, where English was not their first language, people with health anxiety and people who shared medical advice with a relative.

There was one where the patient did not speak English as a first language […] she felt that was going to be really helpful to have that written that she could go over afterwards […] and then I think one patient I used it in was an elderly patient and she wanted me to send it to her daughter so her daughter knew what was going on, so that was really useful. (GP 6, Intervention Development, Round 3)

One GP explained how she thought the report worked well on patients with health anxiety by removing uncertainty:

A lot of [health] anxiety comes from uncertainty so if you take away that uncertainty by laying out what the plan is, I think that’s helpful for a patient with health-anxiety. (GP5, Intervention Development, Round 3)

Patients who received the report explained why they felt it was useful for them:

I suffer from dyslexia so I kind of forget stuff quite quickly and having it on my phone; I remember her name and everything. (Patient 18, Intervention Development, Round 3)

I have some memory issues and I put that down to be a bit ancient but it’s nice to have the piece of paper because and then she says, you know, what we discussed and what I was able to raise and about what happens next, which is really helpful. (Patient 33, Feasibility Study)

These patients confirmed, without prompting, that the GPs had been correct in selecting them based on their own personal characteristics.

##### 3.2.4.3 In what types of consultations

The summary report was most useful when the consultation was complex and the follow-up required multiple steps.

the patients I have used it on, I have given them multiple steps […] they were complicated, ‘we are gonna start this medication then you will need a blood test in two weeks and then you will need to have an X-ray or do a referral’ […] I think that’s very difficult for a patient […] especially if there is some bad news in there, it’s difficult for them to remember everything, so the patients I asked would you like it in written form, they were all very keen. (GP 6, Intervention Development, Round 3)

This GP used the form when there were multiple steps, especially if there was bad news which the patient might find it difficult to process or remember. Patients confirmed that the reports were most useful in complex consultations and would not be useful for simple consultations:

If I just went and said, ‘I’ve got a bit of a cold’ and they said, ‘I’ll prescribe you some Aspirin’, I don’t think I’d be bothered about having a report for anything more trivial or minor. (Patient 13, Intervention Development, Site 1)

GPs and patients also felt the report was useful when the complex consultation was over the telephone:

In the past, if I was seeing a patient I might write on their own paper for them, if I was concerned about memory, I might get a scrap piece of paper even and write out the plan of action for them, but when you are not seeing patients face-to-face sometimes and it’s just a telephone call or whatever, you don’t have that resource (GP2, Intervention Development, Round 1)

However, GPs pointed out that, since COVID-19, they increasingly text patients. For a simple instruction it is much easier to use an SMS message rather than the summary report template.

The other two elements that worked well are discussed within the individual CMOCs they apply to.

#### 3.2.5 Context-Mechanism-Outcome configurations (CMOCs) Patient and family clear on safety-netting and follow-up required

*CMOC1:*

When GPs are aware of the need to clearly communicate complicated follow-up to patients and write a tailored report in patient language (C) the patient and their family are clearer on the follow-up required (O) because the GP reflects more on how to plan and communicate this follow-up while they are completing the report (M). (Box 1)

Some GPs said the structured template made them reflect more on planning and communicating the follow-up:

It makes you think actually you know what have I explained to the patient? Do they understand what the next steps are? I think we always feel that we’re doing that but you know perhaps the patient isn’t quite clear. So to actually lay it out and say look, this is the plan. I will do this, I will phone you about this, you need to do this, you know these results will be back in X days is actually really helpful (GP5, intervention development round 3)

I think it made me probably be a bit more specific about my safety netting […] I actually never left the generic message […] because they are going to show it potentially to relatives and it needs to be not really open to interpretation. (GP6, intervention development round 3)

The first GP felt that completing the report made them more careful about how they communicated within the consultation. The second GP said that, knowing the report might be referred to and shared with relatives, made her more specific about her safety netting advice, listing specific signs and symptoms to look out for.

Provided the report was written in clear language, it gave patients and their family clarity on the follow-up required. Some patients said that without the summary report they would not have been aware (or would not have remembered) this follow-up:

[The consultation summary] said a review would need to be booked to deal with the new start of this tablet that I’ve just started on Friday […]So it gave me some information of what I would need to now do with the start of this new one, otherwise I probably wouldn’t have known that I had to book a review in four weeks to see how I’m feeling and to see if it needs to be increased. (**Patient 19, Intervention Development, Round 3**)

Other patients also described how they were clear on what to do next and what to expect from the GP.

*CMOC2:*

When a patient who has poor memory or likes to share medical information with family has had a consultation which is complex or involves multiple follow up steps receives a bulleted easy-read report soon after the consultation (C) the patient and their family are clearer on the follow-up required (O) because the patient has a memory aid to refer to or to share with their family (M) (Box 2)

The majority of interviewed patients said that the report functioned as a memory aid:

it’s still useful because – especially at my age – you don’t always remember, and still useful to have that to fall back on. (Patient 24, Feasibility)

she gave me quite a lot of information over the telephone, had it not been in a report I probably wouldn’t have remembered all of it. **(**Patient 17, Intervention Development round 2)

If you’ve not been able to quickly write everything down on a piece of paper, which I try to do, you’ve got it in a text message, so you can go back to it and you can see what you need to download or what the next step is to do with your health. (Patient 19, Intervention Development round 3)

sometimes when you’re getting information in medical terms and in a hurry it can be a bit difficult to remember everything so I think the printed report is excellent. Patient 33, Feasibility

you think you can remember everything they’ve said and even when you’re younger sometimes you can’t remember everything or the intonation is such that you think, well you said such and such a thing, well no I didn’t. So it’s quite useful either to have a rough copy of what you’ve said (Patient 40, Feasibility)

The quotes show different types of patient using the report as a memory aid. Patient 24 was an older patient whose memory was failing. Patient 17 had received complex information over the phone the summary helped her remember it. Patient 33 referred back to the specific medication advice a month later to verify it. Patient 40 was a younger patient without memory problems but felt that the context and intonation used by the GP in the consultation sometimes made her mis-hear or misinterpret what was said. Receiving a written report clarified the content of the conversation for her.

For the summary to serve as a memory aid, the immediacy of the report was important to patients:

It arrived the same day, which is the immediacy of it. Because it was still fresh in our minds what had been going on. (Patient 14, intervention development study, round 2, received report for elderly relative)

GPs felt the report would help patients remember when a lot of information was provided:

I just think people forget, we give them too much information in our ten-minute slots and we think we know what we have said but actually either sometimes we possibly forget to say it and think we have said it ourselves or the patient can’t take all that much in. (GP3, Intervention development study, round 2)

One GP explained that her first line of management is often a “wait and see” approach where the patient is advised to reconsult if symptoms persist after a specified duration. This GP said patients often perceive this as no action taken, and either forget or do not process the advice. The summary report reminds these patients of the safety-netting plan and timings.

Some patients found it beneficial to share the report with family members. One interviewee attended the appointment with her father and received the summary report on his behalf.

If someone tells me something, I might remember some of it but if it’s written down and I can refer back to it, then that’s far easier. Far easier and with my father, it means we can take it home and then discuss it when he’s less tired and able to take it in.(Patient 14, Intervention Development Study, round 1)

The interviewee found it easier to discuss the information with her father when he was more relaxed and ready to process the information. This was particularly relevant during the UK COVID-19 lockdowns when families were unable to meet face-to-face. The bulleted formatting enabled a point-by-point discussion facilitating this conversation between patients and their relatives / carers. One GP explained that it helped her engage more with a patient when she previously might have had to engage with his spouse.

One of them had a spouse with them, but it just made me feel that rather than deal with the spouse for speed […] it helped me just balance, well that patient can think about it again if something is written down, so whereas I would have used the spouse as a safety net, I actually felt I was keeping some empowering still of a patient whose memory was not that good. (GP 2, Intervention Development Study, round 2)

Without the summary report, this GP might have dealt directly with the spouse to ensure that patient’s safety. However, despite having a poor memory, the patient was able to understand health information if given space to process at his own speed, so the summary report was the GPs way of enabling him to take some charge of his own health.

##### Patient reassured

*CMOC3:*

When a bulleted easy-read report is given or sent to patient soon after consultation and this report reflects the patient’s recollection of the consultation (C) the patient is reassured (O) because the information provided is the same as what they thought would happen (M) (Box 3)

Patients found the consultation summary reassuring when it concorded with their recollection of the consultation. Two patients described this as follows:

It was really helpful, well reassuring, wherever the word might be, to get that written down. (Patient 28, Feasibility)

I think sometimes as I say, there’s so much fear going on as I say with COVID as well, I think people’s mind might be in overdrive so any reassurance for any ailment nowadays is nice so that you know that what you’ve heard is right. (Patient 17, Intervention Development, Round 2)

GPs also felt that some of their patients found the summary report reassuring and had selected some patients on this basis.

It’s reassuring for them for me to say thank you for phoning today, this was our plan […] they’ve got it there in black and white what the plan is and what the timescales are. So they’re not thinking oh my gosh, Dr **(Name)** hasn’t phoned me back yet. (GP 5, Intervention development study, round 3)

This same GP said patients often called the reception for test results before they were ready. She felt having the summary in writing would reassure patients about the plan that had been agreed and they would be more likely to wait the specified time before following up. Another GP agreed with this and felt that this reassurance was particularly useful when the time-frame was weeks or longer. One GP described a three-week timeframe:

I wrote out quite clearly that you will need to give your nail clippings to us, pick up an envelope from the surgery, they then get sent off, it will take three weeks at least for the results to come back so don’t expect anything until then. So it was – and I think that’s really helpful to give those timeframes because then they’ll stop keep phoning up to find out where is my test results. (GP 7, Feasibility Study)

This GP had seen a patient with a fungal nail infection who needed to have his nail clippings sent for investigation. The GP believed that having the timescales in writing would reassure the patient about the expected timescales and lead to less phone calls to the practice.

##### Increased patient knowledge and empowerment

*CMOC 4:*

When patients are clear on the follow-up and safety netting outlined in the written report (Outcomes -> Contexts), the patient is empowered and their knowledge is increased (O) because the patient knows what they and the health care professional are responsible for and need to do (M) (Box 4)

Some GPs felt they were able to empower patients by providing them with an agreed written plan which the patient could then execute, particularly when this plan was largely the patient’s responsibility. One GP described a patient who had been advised to make lifestyle changes.

The outcomes and plans relied heavily on him making changes to his lifestyle […] there’s six things he needed to do and to have that written down on his phone I think is really useful so he can go back and say ‘look, this is what I said, this is what we discussed and this is what we’ve agreed to try to do’ and I think, um, yeah, I think that’s great actually […] I think they’re going to take it more seriously and really see it as something a bit more real than just saying ‘you should go for a walk each morning’. (GP 9, Feasibility Study)

This GP felt that, having a bulleted list of six actions the patient needed to take would empower him to action this much more than if the information had been conveyed verbally. The GP described this as the patient “taking it more seriously.” Patients also felt that having the information in writing enabled them to execute it more easily. One patient described the report as creating a “target”:

You can follow up and you can challenge it and I think people are happy with that and say well, you said you were going to do something in two weeks and it hasn’t happened so it does set a little target for both parties in a way which at the moment is quite useful. (Patient 33, Feasibility Study)

This patient saw the document as a contract her and her GP, which she felt empowered to follow-up and challenge.

*CMOC5:*

When GPs discusses / co-creates the report with the patient (C) the patient is empowered and their knowledge is increased (O) because the GP and patient have agreed the shared plan together (M) (Box 5)

Some GPs discussed the report with the patient as they generated it in the consultation. One GP explained that she purposely used the language of shared decision-making in the summary in order to emphasise that it was a jointly agreed plan:

I kind of made it a shared care. I mean I think it is a shared care but rather than a kind of paternal, ‘this is what I’m doing’, I made it, you know ‘Diane thank you for phoning today and this is what we discussed and this is our plan’ rather than you know, ‘this is my plan’ as a doctor. So I think it’s a nice way to get the patient to feel involved in their care […] That’s how I chose to interpret it but you know, I think everyone will do their own thing with it. (GP 5, Intervention Development Study, Round 3)

This GP often read the report to patients in the consultation to empower the patient to take ownership of the plan. The GP suggested that this might even improve her communication when she was not completing the report:

I think even when I’m not using the intervention I might summarise more at the end now having used it to say, okay so let’s just recap our plan that we’ve made together and I think it’s very much the, we’re making this plan together and that comes across I think in that COAC sheet. (GP 5, Intervention Development Study, Round 3)

The COAC report gave this GP a mechanism for emphasising that the plan was a shared one they had made together, and she could potentially employ some of the same techniques even when not generating the report.

One patient described how the GP went through the report with her beforehand:

she said ‘well this is what we’ve talked about, this is what I’ve recommended, these are my suggestions’. She went through it with me, she didn’t just hand it to me and it wasn’t verbose in any way, it was quite brief, but it was totally to the point and yeah, and useful. (Patient 40, Feasibility Study)

This patient valued that the GP reviewed the report with her beforehand and referred to the GPs advice as “recommendations” and “suggestions”.

*CMOC6:*

When GPs complete the EMIS template with sufficient detail in patient language and the report that is shared with the patient contains new or missing information or something they did not remember from the consultation (C), the patient is empowered and their knowledge is increased (O) because the patient can look up medical terms or seek clarification from the GP (M) (Box 6)

On some occasions the report either had something missing, or an additional element that the patient did not recall from the consultation. This meant that the patient could follow-this up, which increased their knowledge and empowerment. One patient had attended a consultation with her father. The report contained information about the tests and referrals but did not mention a form she had been expecting. This flagged that the GP had probably forgotten so she followed this up:

from memory I think there was something missing that I thought we had discussed […] It was about a respect form. You know, the ‘do not resuscitate’ forms. That we discussed and I haven’t seen since. It wasn’t on that message. I’m just going to follow that up. I’m able to do that because I’ve seen that gap whereas I wouldn’t have known that otherwise [….] She agreed that she would get one printed and in the post to him, which he’s not had. That’s fine, we’ll follow that up. (Patient 14, Intervention Development Round 1 – received report for elderly relative)

This patient felt that, had she not received the consultation summary a few hours after the consultation, when it was still fresh in her mind, she would have forgotten about the GPs promise to send a DNR form, but because she noticed the gap in the summary it reminded her to follow this up.

Some patients were able to look up information online as a result of receiving the report. One patient explained how he had a greater understanding of his cholesterol levels:

When she said it to me, that she was putting me on tablets, I’d already got my mind made up that the number (cholesterol level) must be really high to be put on tablets for it but when I looked at it in writing I thought, it’s [only] slightly high. […] Then I get on the computer and look at what is normal and what my number was, so yeah, it was useful for that […] It was great, it opened my eyes that it wasn’t as serious as what I thought. I thought I had to go rush out popping pills. (Patient 20, Intervention Development, Round 3)

This patient had assumed that because he was prescribed medication that his cholesterol level was very high. When he received the consultation summary he realised that the GP had only written “slightly high”. He then looked up the levels on the internet which confirmed this and he was able to use this information to inform his decision about taking medication for his cholesterol.

##### Audit Trail

*CMOC 7:*

When GPs write the report with sufficient detail and it is saved to the medical record (C) this provides an audit-trail for medicolegal purposes (O) because the report can be referred to in case of a legal dispute (M) (Box 7)

Some GPs thought that the summary report might be useful for future for medico-legal purposes:

I think medico-legally it would be better as well because you can really go look I did tell them that, there it is in black and white and they have been sent it, they have had this document, they can’t say they didn’t know because I sent it to them. (GP 3, Intervention Development, Round 2)

This GP felt that having the summary report saved to the patient record provided demonstrable proof of the advice given to the patient. In counterpoint to this, another GP suggested that if not well completed, the summary report could *expose* the practice medico-legally.

I guess there’s some slight nervousness if you’re putting very specific safety netting down have you got everything if you know what I mean. If there’s something you miss then are you potentially in more trouble. If you don’t put those specific safety netting then how useful is it. (GP 1, Intervention Development, Round 1)

This GP was referring to the safety-netting advice, where GPs can tick the generic statement *“Contact the surgery if your symptoms get worse or you develop any other symptoms of concern as we discussed. When we are closed you can call 111 to speak to an Out of Hours GP.”* GPs were encouraged in the training to add more specific advice to this for example by listing the potential symptoms of concern. This GP was concerned that by adding these symptoms of concern the GP would be exposed if one symptom was missing.

##### More appropriate care pathway for patient

*CMOC8:*

When GPs are aware of the need to clearly communicate complicated follow-up to patients and write a tailored report in patient language (C) this can lead to a more appropriate care pathway for the patient (O) because the GP reflects more on the follow-up while they are completing the report (M) (Box 8)

Some GPs explained that, as well as helping the patients, the process helped their own thinking and made them clarify why they were taking particular actions.

It acts as a prompt for me. To think about what I need to do. To make sure this patient is getting what they need. Makes me think why I’m doing it and when I’m going to do it and it. (GP 4, Intervention Development Study, Round 2)

Sometimes we are just screening and if the test is negative there is nothing to be done, but sometimes we are screening in a way that a negative test actually means more action is needed […] so it just clarified when the review was going to be and make you make a decision about what action was beforehand, whereas sometimes I might leave that open in my head. […] it’s not a bad thing to try and organise your thoughts for anything you might have done. (GP 2, Intervention Development Study, Round 1)

These GPs explained that the summary report made them reflect on this in advance. The second GP considered the reasons for ordering the tests more and the likely next steps for the patient.

Some patients also felt that the summary report had led their GP to reflect more on the follow-up which led to a more “joined-up” care pathway.

And then in two weeks’ time I’m going to do another stool sample, and go in again to have some blood’s at the same time. So, it was very good because it was almost like, wow, we’ll do that at the same time, and it was just like, oh God, isn’t this lovely? I’ll take in a sample, she said, when you bring that in we’ll just do the bloods at the same time and it’s just like, oh, that’s nice and joined up, isn’t it? (Patient 28, Feasibility)

The patient attributed the appointments being booked at the same time to planning and communication which was necessary to create the summary report. This may not be the case, as since the COVID-19 pandemic some GP practices are ensuring appointments coincide to reduce footfall in the practice^21^.

*CMOC9:*

When GPs write the report clearly, with sufficient detail, in patient language and the report and codes are saved to EMIS (C), this can lead to a more appropriate care pathway for the patient (O) because other GPs can refer to the report to see what was agreed (M) (Box 9)

Some GPs felt that the summary report could improve communication between GPs by providing a record of what was communicated to the patient.

If there’s a recurrence or a flare-up or an ambiguous test result comes in that they [another GP] see on my behalf so to speak, then being able to see what the patient knows [is useful…]. it’s not just in our head but it’s actually recorded in a way that other people [clinicians] can more easily track where we’re at with managing the patient. (GP2, Feasibility Study)

This GP felt that the summary report would improve management continuity of care in practices where relationship continuity was difficult to maintain. One GP said she sometimes wrote paper notes for patients. While this might help the patient, it did not improve their management continuity:

I used to scribble it on a bit of paper, there is no audit trail with that, once it’s given to the patient it’s lost, the next GP doesn’t see it, the family may never see it, I think having it written in an audit trail that appears in the patients notes is just fantastic. (GP 6, Intervention Development Study, Round 3)

Although the summary report could improve management continuity, it could have the opposite effect in the short-term if all GPs were not familiar with it:

In large group practices, other people are reading our notes a lot of the time and if you go out of your normal way of writing down history and follow up, the potential that somebody else who is quickly trying to retrieve information might not spot it so easily. […] If a whole practice wasn’t familiar with it, I can imagine they might get confused. (GP 2, Intervention Development, Round 1)

This GP’s reflection highlights that the summary report would be best implemented as a practice-wide intervention if it is to improve communication between GPs.

## 4 Discussion

### 4.1 Main findings

This paper reports on the person-based development of a consultation summary report and on a realist evaluation of this which was embedded within a feasibility study. The person-based development was highly successful. Numerous improvements were made to the summary report and GPs and patients agreed the final version was much improved on the initial version.

In the feasibility study the summary report was tested in a single intervention with a pre-consultation form. Through the embedded realist evaluation, we found that these were useful for different types of patient. The summary report is most useful for patients who have been given complicated follow-up and might not remember this follow up. It was only useful to patients if GPs completed it with enough detail. Although many patients felt that it would be useful to receive the report routinely, GPs felt it wouldn’t be sufficiently useful to patients to justify the time completing it in situations where patients did not have complex follow-up. Patients felt it was less useful when it was not competed with enough detail.

We identified five possible outcomes of the summary report which are captured in our finalised programme theory. The three outcomes with the most qualitative evidence were: 1) patients and family clear on follow up provided; 2) greater patient knowledge and empowerment and; 3) patient reassurance. There was also evidence that the summary report might help medico-legally and lead to more appropriate care pathways for patients because all practice staff can refer to the summary report to see what was agreed, although there were also concerns that the report could expose practices medico-legally.

### 4.2 Strengths and limitations

The person-based approach was an effective method of developing the consultation summary report. The PPI group were actively engaged and an important part of designing of the intervention. Practices were effectively re-engaged following the COVID-19 related study pause. An effective collaboration was developed with the GP Federation for BNSSG CCG (One Care) who were able to publish the required EMIS resources to the GP practices.

The realist evaluation is a well-established theory-based approach for making sense of why, when and for whom context-sensitive outcomes occur in complex interventions, such as the summary report. We analysed data across 32 interviews and used rigourous methods to analyse this data within a 3-person team. We had fewer patient interviews than planned across each phase. However, there was a high degree of concordance among patients on the use of the summary report and therefore we did have enough information power to develop the programme theory and have included additional quotes to demonstrate that the findings are grounded in the data. We also carried out more extensive PPI in the intervention development phase of the summary report to compensate for the lack of patient interviews.

### 4.3 Extended use of patient and public involvement (PPI)

Patient and public involvement was an essential part of developing the summary report. The PPI group shaped and refined the design of the pilot report in two meetings. Further to this, because of the difficulty we had eliciting wording preferences from our interviewees, we carried out an extended PPI consultation exercise on the wording of the standard text used in the template.

PPI differs from qualitative research in that the latter seeks to address questions relating to “why?”, “how?” and, “for whom?^22^ through collection and analysis of qualitative data, whereas PPI is used to improve the design and conduct of research, rather than providing data to answer research questions. PPI in research is carried out *with* or *by* members of the public and is a key part of the research process^23,24^.

In the case of our wording-preference elicitation, the input of the PPI group overlapped into qualitative research. Unlike in pure qualitative research, our PPI group were engaged in the analysis and interpretation of data which they themselves had provided. The suggested structure, ‘look and feel’ of the report and much of the wording was generated or refined through PPI meetings. The PPI group generated data (preferred wording); this was analysed by the researcher and the outputs (wording choices) further considered and tested by the same group in later meetings.

This approach worked well for our study design. The critical importance of using PPI in all elements of the process of developing, testing, evaluating and implementing complex interventions is explained by Richards *et al*.^25^. The developers of the person-based approach advocate combining PPI with qualitative research to generate a diversity of feedback, which can create more engaging interventions than would have been possible to achieve through PPI or qualitative approaches alone^26^.

Other studies have usefully combined PPI when developing complex interventions, for example Best et al used PPI contributors to identify themes arising from qualitative data collected during complex interventions^27^ and Morgan et al found combining PPI with qualitative research allowed inclusion of a wider range of views than would have been possible with qualitative research alone^28^. Mann et al found that PPI positively affected a the design and implementation of a large randomised controlled trial, including changes to documents used in the trial and advice on qualitative data collection methods and analysis^29^.

### 4.4 Summary report comparison with literature

There is little literature on producing a summary report for patients in UK primary care. A 2020 realist evaluation of discharge letters in a *secondary* care setting showed that, if patients understand their letters this can lead to patient empowerment and improved patient knowledge and recall^30^. However, hospital discharge letters are primarily for the GP, although the patient may be sent a copy. They therefore contain medical jargon and acronyms. Some patients and GPs agreed that the patient would have benefitted from use of lay terms in the letter^30^.

An important feature of the summary report is the documentation of safety-netting advice. A 2021 study of 295 GP consultations showed that over two-thirds involved spoken safety-netting advice, but that this advice was documented in only one third. The practice of GPs varied widely, from those that did not document their safety-netting advice to those that nearly always did so. The authors suggested this lack of documentation could have medico-legal complications in the event of an untoward incident^31^. Use of the COAC summary report would improve this documentation in the case of complex patients and consultations and the interviewed GPs suggested this might have medico-legal uses, although one GP pointed out it could have adverse medico-legal consequences if safety-netting advice was missed. In the US, failure to notify patients of test results has been identified as a relatively common occurrence, particularly when there is not a systematic process identified for doing this^32,33^. GPs commented that the COAC summary report helped them to reflect on the follow-up, and patients that it gave them an agreed record of this follow up, which should help with future notification of test results. The literature shows that review templates for long-term conditions can improve documentation and act as a reminder but have sometimes acted as a barrier to provision of patient centred care^34^. There was no evidence that this happened in this case; indeed both patients and GPs thought the template helped make the consultation more patient-centred, perhaps because it was selected for patients with complex consultations who were most likely to benefit from it. Some GPs changed their consultation style to write the report with the patient, sometimes asking the patient to repeat back to them. This kind of “teach-back” has been found to be effective across a wide range of settings, populations and outcome measures^35^. The pandemic has shown the value of GPs working together within primary care networks (PCNs). Practices are merging and the average practice size increasing; patients both seeing and having a preferred GP has reduced over recent years as relational continuity has consequently declined^36^. Putting what was said to the patient on a record which can be shared among GPs may be one simple way to improve informational continuity within general practice.

#### Sharing information through the medical record

Some patients thought the summary report should be made available through the medical record via the “patient access” portal. UK primary care patients have, in theory, had access to their medical record through this portal since 2015. The government committed that all patients would have access by 2018^37^, but in a 2020 study on patient access to the medical record, only 18.3% of patients used this access to view their medical record. 75% of these users placed a high value on the access. The study authors noted that the low levels of access may be due to the permissions of the GP practice rather than the preferences of patients^38^. GPs have previously expressed concerns that patient access to records could increase risks of litigation and require them to change the way they write on the records so that patients can understand them^39^. Patients have expressed a need for support and training in using and understanding the online record^40^. A systematic review of patient record sharing in UK secondary care found that the approach of giving information to patients almost exclusively verbally was insufficient; and patients should have access to notes, but that simply allowing full access, without explanation or summary, is also insufficient^41^. The COAC summary report may facilitate this shift in recording information in the medical record as it allows a patient-friendly report to be generated and stored alongside more technical notes. As the RCGP guidance on medical record sharing states, writing certain sections of the medical record more clearly and in lay terms will be of benefit to both patients and clinicians, but technical medical information is often necessary for patient safety^42^.

There is evidence that sharing information with patients through their medical record can lead to some of the outcomes we identified in our programme theory. For example, a US-based study found that sharing notes with patients helped engage them to improve record accuracy and health care safety together with practitioners^43^. We similarly noted that sharing the summary report led to conversations with the patient and GP following the consultation to clarify the advice. Educating patients with timely medical information through their smartphones or tablets has been shown to improve their levels of knowledge, medication or treatment adherence, satisfaction, and clinical outcomes^44^. This resonates with our qualitative findings that the summary report improves knowledge and patient empowerment. Although we did not have sufficient qualitative evidence to suggest clinical outcomes would be improved, it is entirely possible that they would be over a period of time.

### 4.5 Conclusions

The summary report was developed and tested using rigourous methods and has been demonstrated to be valuable for both patients and GPs. It appears to be particularly useful for patients who have been provided with a lot of information including important follow-up or safety netting. For these patients, it provides a mechanism for remembering the follow-up, leading to reassurance and patient empowerment. It may also improve the care pathway and the audit trail for medico-legal purposes. It requires a few minutes of GP time per consultation, so from the GP’s perspective it would be important to select the patients who were most likely to benefit to ensure this trade-off is worth it.

## Supplementary Material

Supplementary Information

## Figures and Tables

**Table 1:** Coding framework for Table of Changes.

Coding framework			
Code	Stands for	Means
**IMP**	Important for intervention uptake and effectiveness	This is an important change that is likely to impact intervention uptake or effectiveness or is a precursor to that (e.g. acceptability, feasibility, persuasiveness, motivation, engagement), and/or is in line with the Logic Model, and/or is in line with the Guiding Principles.
**EAS**	Easy and uncontroversial	An easy and feasible change that doesn’t involve any major design changes. For example, a participant was unsure of a technical term, so you add a definition.
**REP**	Repeatedly	This was said repeatedly, by more than one participant.
**EXP**	Experience	This is supported by experience, for example: PPIs agree this would be an appropriate change. Experts (e.g. clinicians on your development team) agree that this would be an appropriate change. Literature: This is supported by evidence in the literature.
**NCON**	Does not contradict	This does not contradict experience (e.g. evidence), or the Logic Model, or the Guiding Principles
**RES**	Research relevant	This is a change to the design of the research, not the intervention
**NC**	Not changed	It was decided not to make this change. Please explain why (e.g. it would not be feasible; or only one person said this).

**Table 2:** Patient recruitment target in control and intervention practices.

	Intervention	Control	Total
**Practices**	4	2	6
**Patients (pre-consultation)**	120	60	180
**Patients (closure report – no target set 30% estimate)**	36		36

**Table 3:** Development of the pre-consultation form: GP and patient recruits.

Pre-consult form	Practice IMD Score	Date	Recruiting GPs	Patients recruited per practice (target = 15)	Patients interviewed per practice (target = 6 to 7)
Practice 1	9	Nov 20	2	14	3
Practice 2	5	Jan 21	2	14	2
Practice 3	1	Mar 21	2	15	3
Total			6	43	8

**Table 4:** Guiding principles – consultation summary report.

Intervention Design Objectives	Key Features
To make the summary report easy for GPs to complete	▪ Easy to navigate to in EMIS (minimum number of clicks) ▪ Can be done within the time of a normal consultation ▪ Sense of it being integrated with EMIS ▪ Visually appealing
To create a positive and beneficial experience for GPs using the report	▪ Seems relevant to patient from GP point of view ▪ Access and use of the template fits within normal process of the consultation
To make the report as useful as possible for patients	▪ Seems relevant to patient ▪ Ensuring is clear to the patient ▪ Easy for patients to access ▪ Ensuring the intervention provides something interesting, relevant, and helpful for the user (patients)

**Table 5:** Summary of changes: consultation summary report.

	Issue identified	Feature added
**External Issue**	The initial intervention was conceived for face-to-face appointments including printed advice. After March 2020 most appointments were by telephone so this wasn’t possible.	Changed the process design and the user guides so that the report could be either printed, sent via accuRX or email.
**PPI/Patients**	The original version of the summary report had four sections. The PPI group found this visually confusing and suggested combining sections. Patients subsequently suggested dropping the final section of “problems raised today”.	Report reduced to two sections: ▪ Plans and advice from today ▪ Next steps
**PPI / Patients / GPs**	Some GPs thought the report should directly address the patient from the GP. Because of the low number of patient interviews, we ran a two-step PPI process: a) we elicited feedback on the wording through a PPI meeting b) based on this feedback, we presented PPI members with options to select their preferred wording from.	Extensive wording changes were made to make the language more patient-centred (e.g. stool was changes to “stool (poo)” and to make the report address the patient from the GP: (e.g. “contact our reception team” instead of “contact your GP reception” and “I have referred you” instead of “we have referred you”
**Patient**	The original report sent to patients was difficult to read as it was in paragraphs. Although most of them found it useful, patients felt it would be easier to read and to talk to their carers about if it was a bulleted list.	We were aware of this issue early on but it was technically difficult to solve. A solution was devised in Round 3, which involved GPs installing a bespoke macro to format the report once it was generated.
**GP / Patient**	Where medication changes are indicated these were not clear.	Added an optional section for medication changes.
**GP**	The initial version of the EMIS protocol used three templates. This required the GP to select in advance whether they were ordering “no tests”, “one test” or “two tests”. The two-test version was heavily free text based.	A single template was developed, no matter how many tests were ordered with mainly tick box questions throughout. This also relied on development of the macro which GPs installed on their computers.
**GP**	There was only one box for advice to patient. Some GPs ran out of space and wanted to put advice each problem on a separate line.	Adjusted so that there are three advice boxes. Only the first is compulsory to complete.
**GP**	The original version had default text for booking tests which GPs overwrite e.g. “contact reception to book your X test”. GPs found they were always overwriting the same things, so suggested the text default to a standard.	Put the most common ways of booking tests e.g. “please bring in a sample for your urine test”, “phone 0141 332 4345 to book an X-ray appointment.” This was customised for each practice.
**GP**	The template works by writing codes into the patient notes. Another clinician reading the notes may be confused by this.	All COAC clinical codes written to *comments* section of EMIS. A research code is added with text: *“This patient participated in the COAC Study. These codes were used to create a report which is attached to the record below.”*
**GP**	Would be useful to indicate use the template for fast-track referrals.	Added fast-track referral text
**GP**	Would be useful to direct patients that the NHS app is another option for viewing test results	Add optional tick box “you can also view your results on the NHS app”
**GP**	In the training sessions, GPs noted that some of the free text did not apply to policy in their practice (e.g. phone number for referrals).	Customise the free text in each box for the practice policy after the GP training session.

**Table 6:** Patient and practice interviewees for the summary report.

	Intervention Development	Feasibility Study
***Patients***		
Site 1	*3*	-
Site 2	*2*	*4*
Site 3		*6*
Site 4		*1*
Site 5		
Site 6	*3*	
***GPs***		
Site 1	*2*	*2*
Site 2	*2*	*2*
Site 3		*2*
Site 4		*1*
Site 5		
Site 6	*2*	
**Total**	***14***	***18***

* Sites 1, 2 and 6 were in the Intervention Development Study as well as Feasibility. Sites 1 to 4 were intervention sites and sites 5 and 6 were control sites.

## Data Availability

**Underlying data** Researchers can apply for this data via a form on the repository: University of Bristol: COAC Study Qualitative Dataset, https://doi.org/10.5523/bris.1ljvagu1sigje2duqj3ube527y (restricted access)^45^. This project contains the qualitative data transcripts for the COAC Feasibility Study, where participants agreed that these could be shared with bona fide researchers outside the Bristol research team. Information about each transcript is listed below, as follows: Transcript ID: The name of the transcript in the folder. The name consists of: * a participant identifier * the type of participant (patient, clinician or administrator) * the site (1 to 4 – this was not reported in the paper for reasons of anonymity) * The date of the interview Participant identifier used in papers: This is the identifier used in this paper. The folder also contains the consent form. All patients in this study consented to point 7 in this form: “I understand that after the study my anonymised data will be made available to bona fide researchers for future research studies, and it will not be possible to identify me from these data. If I agree to this, my data will be held for twenty years.” This dataset has an access level *Restricted*, which means it is not available via direct download but must be requested. Research participants did not give explicit consent to share this data as open data but agreed that it should be made available to approved bona fide researchers only, after their host institution has signed a Data Access Agreement. In order to request access to this data please complete the data request form available from the link above. We will consider any application from any organisation where an established research governance process is in place. Data are available under a Non-Commercial Government Licence for public sector information. **Extended data** University of Bristol: COAC Study Extended Dataset, https://doi.org/10.5523/bris.386dsq2e4iii225ms7du8pd5jq^46^. This project contains the following extended data: - COAC-pre-consultationForm.doc This file contains screenshots of the pre-consultation form which patients responded to in the COAC Study. - COACStudy-pre-consultationform-TableOfChanges.doc This file contains a detailed table of changes made to the pre-consultation form in the COAC Intervention Study. Patients who are quoted in this table all consented to the first six points in the consent form included in this folder. - COACStudy-SummaryReport-TableOfChanges.doc This file contains a detailed table of changes made to the summary report in the COAC Intervention Study. Patients who are quoted in this table all consented to the first six points in the consent form included in this folder. - COACStudy-TopicGuides.doc This file contains the interview topics guides for the COAC Study. - PatientConsent-Interviewsv1.3.doc This is the patient consent form used for the COAC Study - PatientInfoInterviewStudy2vL4.doc This is the patient information leaflet given to patients interviewed for the COAC Study - COREQ checklist - pre-consultation form This is a checklist for the COREQ reporting guidelines which demonstrates how they were following in collecting and analysing data about the pre-consultation form - COREQ checklist - summary report This is a checklist for the COREQ reporting guidelines which demonstrates how they were following in collecting and analysing data about the summary report. **Reporting guidelines** University of Bristol: COAC Study Extended Dataset, https://doi.org/10.5523/bris.386dsq2e4iii225ms7du8pd5jq^46^. This paper has followed the Consolidated criteria for reporting qualitative studies (COREQ) checklist^47^. The realist evaluation also followed the RAMESES II reporting standards for realist evaluations^48^. See extended data^46^ for checklists. Data are available under the terms of the Creative Commons Attribution 4.0 International license (CC-BY 4.0).

## Box 1 CMOC 1 diagram

GP is aware of the need to clearly communicate complicated follow up and….

## Box 2 CMOC 2 diagram

Patient has poor memory or likes to share medical information with family or consultation has been complex / involve multiple follow up steps and…

## Box 3 CMOC 3 diagram

Report reflects patient/s recollection of consultation and..

## Box 8 CMOC 8 diagram

GP is aware of the need to clearly communicate complicated follow up and….

## Plain english summary

### The problem

Patients sometimes feel GP consultations are too short. Sometimes patients’ problems are missed. At the end of the consultation it can feel rushed and patients may not remember everything the GP tells them. We wanted to improve GP consultations.

### What we did

We developed a way for GPs to create a one-page summary of what was discussed if the consultation was complex. The GP prints this summary for the patient or sends it by email or text message.

### How we tested it

We piloted the summary in three GP practices in turn. We interviewed patients, GPs and an administrator in each practice and made changes based on their suggestions, and on public contributors’ opinions. Each improved version was tested in a new practice. We then tested the final summary report in four practices. Patients who had filled in an online form before their consultation were given a summary after the consultation if their GP thought it would be helpful. We interviewed patients and GPs to find who it was most useful for and when.

### What we found

GPs and patients agreed the final summary report was much better than the first version. Patients found the report extremely valuable. GPs recognised the value but felt it took extra time. It was most useful when patients were given complex advice they might not remember. These patients found it reassuring and empowering to have a summary of the consultation.

### Conclusions

The approach we took was very successful in developing the summary report and patients and GPs found it valuable. As it takes a little longer for GPs to do the summary, it should only be used if there is a lot for patients to remember.
