# Supplementary material for: The person-based development and realist evaluation of a summary report for GP consultations [version 1; peer review: 2 approved]
Source: NIHR Open Res. Author manuscript; Available in PMC 2022 Aug 5. (PMC7613240)
Supplement: Supplementary Information [file EMS143882-supplement-Supplementary_Information.pdf]

Open Peer Review

Current Peer Review Status: ✔ ✔

Version 1

Reviewer Report 14 March 2022

https://doi.org/10.3310/nihropenres.14374.r28504

© 2022 Pettigrew L. This is an open access peer review report distributed under the terms of the Creative Commons Attribution License, which permits unrestricted use, distribution, and reproduction in any medium, provided the original work is properly cited.

✔ **Luisa Pettigrew**
Department of Health Services Research and Policy, London School of Hygiene and Tropical Medicine, London, UK

This is study uses realistic methods, with notable PPI, to develop and test a post-consultation summary for patients generated by the GP through the GP's electronic health record. It is a relatively small study but provides clear realistic methods, with CMOC configurations being presented to build theory in the subject area. The study is particularly valuable as it tests how the GP's electronic health record can be used to generate and share the summary.

The conclusions are adequately supported by the results, where the authors identify the potential benefits of the summary for patients and GPs, as well as identify for which cohort of patients it is most useful and under what circumstances GPs view the additional time needed to complete it as worthwhile. It would be interesting to scale up this intervention to assess how acceptable it is to GPs given the current time pressures of a normal consultation versus the potential benefits.

Is the work clearly and accurately presented and does it cite the current literature?
Yes

Is the study design appropriate and is the work technically sound?
Yes

Are sufficient details of methods and analysis provided to allow replication by others?
Yes

If applicable, is the statistical analysis and its interpretation appropriate?
Not applicable

Are all the source data underlying the results available to ensure full reproducibility?
Yes

Are the conclusions drawn adequately supported by the results?
Yes

Competing Interests: No competing interests were disclosed.

Reviewer Expertise: Primary care & general practice health services research

I confirm that I have read this submission and believe that I have an appropriate level of expertise to confirm that it is of an acceptable scientific standard.

Reviewer Report 14 March 2022

https://doi.org/10.3310/nihropenres.14374.r28502

© 2022 Browne F. This is an open access peer review report distributed under the terms of the Creative Commons Attribution License, which permits unrestricted use, distribution, and reproduction in any medium, provided the original work is properly cited.

Freda Browne
School of Nursing, Midwifery and Health Systems, University College Dublin, Dublin, Ireland

This paper provides a detailed report of the realist evaluation of the development and testing of a GP summary report. It elicited the involvement of key stakeholders (including PPI) in the development of the intervention and the refinement of the summary report. The paper concluded that with select patients the summary report provides for greater patient empowerment and reassurance.

The work is clearly and adequately presented using current literature. The presentation of the study is well supported with tables and figures. There is extensive detail in this paper and the use of the tables and figures helps maintain clarity. The presentation of the CMOC's allows them to be clearly identified. The underlying programme theory is also presented as a figure which allows it to be seen clearly and provides a summary of the findings.

In Background - Rationale for the study - the following sentence is confusing, and I am uncertain as to its relevance here: "*At consultation initiation, sharing the results from patient-reported outcome measures (PROMs) with clinicians can help to elicit concerns*4".

As there are numerous abbreviations used throughout the paper it may be advantageous to also include a list/box of these abbreviations for the reader.

The study design is appropriate and reported clearly. The conventions of realist evaluation are detailed clearly. Realist evaluation is explained clearly for readers who may not be familiar with the design. Sufficient details of methods are included, and supplementary data is available as is source data. Conclusions provided by the paper are supported by the findings and programme theory presented ion the paper.

Is the work clearly and accurately presented and does it cite the current literature?

Yes

Is the study design appropriate and is the work technically sound?
Yes

Are sufficient details of methods and analysis provided to allow replication by others?
Yes

If applicable, is the statistical analysis and its interpretation appropriate?
Not applicable

Are all the source data underlying the results available to ensure full reproducibility?
Yes

Are the conclusions drawn adequately supported by the results?
Yes

Competing Interests: No competing interests were disclosed.

Reviewer Expertise: Realist evaluation

I confirm that I have read this submission and believe that I have an appropriate level of expertise to confirm that it is of an acceptable scientific standard.
